# Association of Childcare Facility Closures With Employment Status of US Women vs Men During the COVID-19 Pandemic

**DOI:** 10.1001/jamahealthforum.2021.1297

**Published:** 2021-06-25

**Authors:** Yevgeniy Feyman, Naomi E. Fener, Kevin N. Griffith

**Affiliations:** 1 Department of Health Law, Policy, and Management, Boston University School of Public Health, Boston, Massachusetts; 2 Corrigan Minehan Heart Center, Massachusetts General Hospital, Boston; 3 Department of Health Policy, Vanderbilt University Medical Center, Nashville, Tennessee

## Abstract

This cross-sectional study assesses the association of closures of childcare facilities with the employment status of women and men with children in the US during the COVID-19 pandemic.

## Introduction

In the US, policy responses to COVID-19 have varied across states, with some states requiring closures of childcare facilities to reduce population mobility and contact among households. Such policies may have been associated with reductions in the growth rate of the pandemic but may also have had unintended consequences. Women perform more unpaid labor than men in couples with children.^1^ Because of these inequities, negative outcomes associated with lack of childcare options, such as reductions in hours worked, may be concentrated among working mothers. A study in Italy^2^ found that the amount of household labor remained greater for women during the early period of the COVID-19 pandemic. We investigated the association between childcare facility closures and employment status among women vs men with children during the first year of the COVID-19 pandemic (January to December 2020) in the US.

## Methods

For this cross-sectional study, we obtained employment and demographic data from the Integrated Public Use Microdata Series, Current Population Survey from January to December 2020. The number of deaths from COVID-19 per month was obtained from the Johns Hopkins Coronavirus Resource Center.^3^ Childcare closure data were obtained from the COVID-19 US State Policy Database.^4^ Our unit of observation was the person-month, restricted to individuals aged 18 to 64 years in households with children. This study was deemed to be exempt from review and informed consent by the Boston University institutional review board because it involved nonhuman subjects research. The study followed the Strengthening the Reporting of Observational Studies in Epidemiology (STROBE) reporting guideline.

We used a triple differences approach with a linear probability model to estimate the association between childcare facility closures and overall changes in employment stratified by sex. Interrupted time series models were then used to assess changes in employment by race/ethnicity, sex, and state childcare facility closure status. Models controlled for individual-level demographic characteristics (eg, age, family size) and state-level COVID-19 case loads, with fixed effects for industry, occupation, month, state, and household. We controlled for race/ethnicity to improve model precision and stratify our findings. We used Stata, version 16-MP (StataCorp LLC) for statistical analysis. Additional information is given in the eMethods in the Supplement.

## Results

This study included 48 920 individuals (13 307 in states with childcare closures and 35 613 in states without closures), of whom 24 452 (49.98%) were women and 24 648 (50.02%) were men; the mean SD age was 43.3 (9.6) years. The sample comprised 165 158 individual-months, including 44 925 individual-months in 15 states that closed childcare facilities by April 2020. All closures were rescinded by June 2020. Employment decreased for both men and women beginning in April 2020 (Figure). Estimates from the triple differences analysis showed that compared with men, the likelihood of a women being employed was −2.6 percentage points (95% CI, −4.3 to −1.0 percentage points) in closure states while closures were in effect. Nationally, this equated to a reduction of approximately 611 000 workplace positions among 23.5 million working mothers.^5^ Estimates were similar when controlling for the number of adults (vs men) in the household and greater when restricting to households with children younger than 6 years (−3.3 percentage points; 95% CI, −6.2 to −0.5% percentage points).

**Figure.**
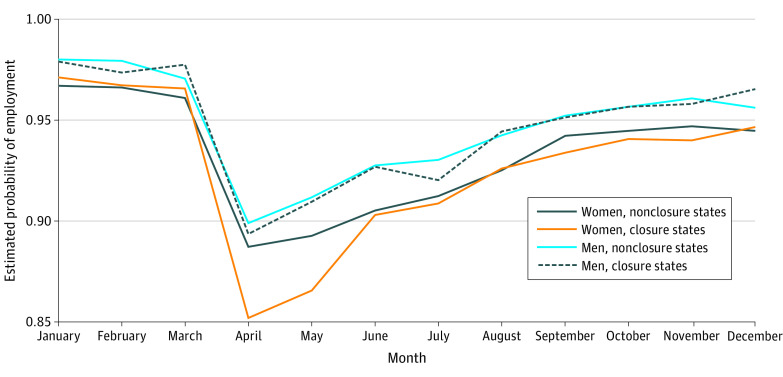
Estimated Probability of Employment During 2020 by Sex and Childcare Facility Closure Status

Interrupted time series models indicated that the largest differences in employment were observed in households with Hispanic and Black individuals, although differential estimates by sex in closure states were greatest in households with Hispanic and White individuals (Table). In a falsification test for households without children, there was no association of sex with employment status (estimate, −0.8 percentage points; 95% CI, −2.2 to 0.4 percentage points), and there was also no association in a test for households with only children of middle school age (11 years to <18 years) (estimate, −1.1 percentage points; 95% CI, −3.5 to 1.3 percentage points).

**Table.** Differential Associations of Childcare Facility Closures With Employment by Race/Ethnicity and Sex

Characteristic	ITS estimate (95% CI)	Individual-months, No.
**No childcare facility closures**		
White individuals		
Men	−0.04 (−0.04 to −0.03)	51 095
Women	−0.06 (−0.07 to −0.05)	48 149
Black individuals		
Men	−0.05 (−0.08 to −0.02)	4366
Women	−0.07 (−0.10 to −0.04)	7020
Hispanic individuals^a^		
Men	−0.06 (−0.07 to −0.04)	11 973
Women	−0.08 (−0.11 to −0.06)	10 884
**Childcare facility closures**		
White individuals		
Men	−0.04 (−0.05 to −0.02)	19 515
Women	−0.08 (−0.10 to −0.07)	19 319
Black individuals		
Men	−0.10 (−0.16 to −0.04)	1663
Women	−0.10 (−0.14 to −0.05)	2596
Hispanic individuals^a^		
Men	−0.06 (−0.11 to −0.02)	2041
Women	−0.12 (−0.19 to −0.06)	2010

Abbreviation: ITS, interrupted time series.

^a^ Hispanic ethnicity was not mutually exclusive with White and Black race.

Estimates from the interrupted time series model for hours worked were greater for both men (−3.9; 95% CI, −4.9 to −3.0) and women (−5.8; 95% CI, −6.5 to −5.0) in closure states than for those in nonclosure states (men: −3.5 [95% CI, −4.2 to −2.8]; women −4.3 [95% CI, −5.2 to −3.5]). Women in closure states had a differential reduction of approximately 1 hour worked unconditional on employment.

## Discussion

In this cross-sectional analysis, state-level childcare facility closures were associated with greater reductions in employment among women compared with men. This association was limited to parents of children younger than 6 years. Our results comport with prior estimates^6^; however, the longer study period allowed us to evaluate whether these associations dissipated by December 2020. We also found that the sex-differential associations were strongest in households with White and Hispanic individuals. In addition, we observed a differential association of sex with hours worked.

Our study has several limitations. First, we could not observe actual childcare service use. Second, because of our observational study design, all findings should be treated as associations. Third, other simultaneously enacted policies (eg, business closures) may have moderated the primary associations, but such policies were not included in this study.

Labor market outcomes may differ by sex, and well-intentioned policies may exacerbate existing inequities. Our findings suggest that additional policy interventions to support women in families with children are needed.

## Notes

Supplement. **eMethods.** Specifications used
