# Supplementary material for: Association of Childcare Facility Closures With Employment Status of US Women vs Men During the COVID-19 Pandemic
Source: JAMA Health Forum. 2021 Jun 25;2(6):e211297. doi: 10.1001/jamahealthforum.2021.1297 (PMC8796877; doi:10.1001/jamahealthforum.2021.1297)
Supplement: Supplement. — eMethods. Specifications used [file jamahealthforum-e211297-s001.pdf]

Supplemental Online Content

Feyman Y, Fener NE, Griffith KN. Association of childcare facility closures with employment status of US women vs men during the COVID-19 pandemic. *JAMA Health Forum.* Published online June 25, 2021. doi:10.1001/jamahealthforum.2021.1297

eMethods. Specifications used

This supplemental material has been provided by the authors to give readers additional information about their work.

© 2021 Feyman Y et al. *JAMA Health Forum.*

eMethods: Specifications used

Triple Differences Specification

While a difference-in-differences specification explicitly requires a parallel trends assumption, a triple differences design does not.[1] The key reasoning is that the difference between two biased difference-in-differences estimators is unbiased so long as the bias is constant in both models. Our primary specification is presented below:

$$y_{it} = \alpha_s + \delta_i + m_t + \beta' X_{it} + \beta_1 Post_{it} + \beta_2 Sex_i + \beta_3 Post_{it} \times Sex_i + \varepsilon_{it}$$

Y_{it} is employment status for individual i at time t, $Post_{it}$ indicates whether the unit is pre or post daycare closure, X_{it} is a vector of time-varying individual characteristics, δ_i is a household fixed effect, and m_t is a month fixed effect. ε_{it} is a serially correlated error term which we cluster within state. Here, the coefficient of interest ϕ is on the interaction of Sex_i and $Post_{it}$ which represents the difference in differences estimate of the effect of Sex on employment. An indicator for closure status was not included as this was captured by state fixed effects. This specification is restricted to individuals between 18 and 64 years old with children in the household.

Covariates in our model included: age, the number of fathers in the household, family size, number of children in the household (not restricted to any age range), marital status, Hispanic ethnicity, race, citizenship status, education level, and monthly state totals for COVID-19 cases. We controlled for changes over time in employment mix with fixed effects for individuals' industry and occupation of most recent employment. Month, state, and household fixed effects were also included with standard errors clustered by state. Individuals were identified as employed if they had a job at time of survey. Individuals' race & ethnicity were self-reported during their responses to the CPS. The list of potential response categories was determined by the U.S. Census Bureau and Bureau of Labor Statistics.

We used IPUMS CPS panel weights to generate nationally representative estimates.

We employed a linear probability model (LPM) to assess changes in our study outcome. LPMs are a special case of ordinary least squares and one of the most popular models in economics and public health.[2,3] The regression coefficients in a LPM represent percentage-point changes in the likelihood of the study outcomes.

We did not consider a propensity-score adjusted specification because parallel pre-trends were clearly visible in Figure 1.

Interrupted Time Series Specification

Our interrupted time series specification stratified by race and ethnicity was estimated similarly to the DDD specification above. However, due to small sample sizes, we removed household fixed effects in this analysis.

The interrupted time series specification for hours worked stratified by sex was performed similarly, but households fixed effects were included. We note, however, that the CPS data on hours worked may be inaccurate, and differential bias in reporting by sex likely biases us towards the null.[4]

Falsification Test

© 2021 Feyman Y et al. *JAMA Health Forum.*

In addition to a triple differences specification (which is itself akin to a falsification test), we explicitly conduct falsification tests by running our primary specification above on households without children, and those with middle school aged children. The intuition is that the coefficient on the interaction term above should be zero if we are capturing strictly the effect of child-focused policies.

REFERENCES

1. Olden A, Møen J. *The Triple Difference Estimator*. Social Science Research Network; 2020. doi:10.2139/ssrn.3582447

2. Lumley T, Diehr P, Emerson S, Chen L. The Importance of the Normality Assumption in Large Public Health Data Sets. *Annual Review of Public Health*. 2002;23(1):151-169. doi:10.1146/annurev.publhealth.23.100901.140546

3. Deke J. *Using the Linear Probability Model to Estimate Impacts on Binary Outcomes in Randomized Controlled Trials*. Mathematica Policy Research; 2014. Accessed April 21, 2021. https://econpapers.repec.org/paper/mprmprres/62a1477e274d429faf7e0c71ba1204b2.htm

4. Harley Frazis, Jay Stewart. *What Can Time-Use Data Tell Us about Hours of Work?* Bureau of Labor Statistics; 2004. https://stats.bls.gov/opub/mlr/2004/12/art1full.pdf

© 2021 Feyman Y et al. *JAMA Health Forum.*
